# Examining the quality of childhood tuberculosis diagnosis in Cambodia: a cross-sectional study

**DOI:** 10.1186/s12889-017-4084-3

**Published:** 2017-03-06

**Authors:** Julia B. Frieze, Rajendra-Prasad Yadav, Khann Sokhan, Song Ngak, Team Bak Khim

**Affiliations:** 1 World Health Organization (WHO), No. 61-64 Preah Norodom Boulevard and Street 306, Phnom Penh, Cambodia; 2 National Centre for Tuberculosis and Leprosy Control (CENAT), Street 278 and 95, Phnom Penh, Cambodia; 3 FHI 360, Street 330, Phnom Penh, Cambodia

**Keywords:** Tuberculosis, Pediatric, Cambodia, Diagnosis, Quality, Clinician, Extrapulmonary, Pulmonary, Low-Income country, Infectious disease epidemiology

## Abstract

**Background:**

Cambodia is one of the 22 countries with the highest TB burden. While childhood TB is estimated to make up 10–20% of total TB cases in high-burden settings, this proportion ranges from 1.3 to 39.4% throughout Cambodia’s provinces, suggesting potential under- and over-diagnosis of childhood TB, subnationally. The proportion of case notifications classified as extrapulmonary TB out of total TB case notifications in children is 87%, greatly exceeding the expected global range of 20–30%. There is a gap in the literature on how childhood TB is diagnosed in resource-poor settings, and the quality of diagnoses. The study’s aim is to quantitatively assess the quality of clinician performance and availability of diagnostic tools, for diagnosing childhood TB in high-burden Operational Districts in Cambodia.

**Methods:**

Between August and September of 2015, a cross-sectional study was conducted at referral hospitals and villages in 24 high-burden Operational Districts. 40 clinicians, and 104 parents whose child was recently diagnosed with TB were interviewed. Questionnaires assessed availability of diagnostic tools, and clinician knowledge and practice during a clinical examination. Descriptive statistics were calculated to provide cross-sectional data.

**Results:**

Availability of advanced diagnostic tools was low. Only 27.5% of clinicians had Xpert machines available at their facility, and 5% had equipment to perform gastric aspiration. 77.5% of clinicians reported that they had a chest X-ray at their facility, but only 34.6% of parents reported that the clinician conducted a chest X-ray. 72.5% of clinicians could name 5 out of 7 main TB screening criteria; however, parent data suggests that clinicians may not be applying knowledge to practice. The mean number of examinations/tests the clinician conducted during the clinical assessment of the child was 1.64. Of the parents whose child had an enlarged lymph node, 60.22% described lymph node characteristics that were not suggestive of TB.

**Conclusion:**

Limited availability of diagnostic tools and suboptimal clinician performance highlight where resources should be allocated to improve quality of diagnoses. Further research needs to be done in low burden Operational Districts to determine the capacity of clinicians and health facilities for diagnosing childhood TB, where cases are likely being missed.

## Background

With an estimated half a million new cases, and 74,000 deaths, annually, childhood tuberculosis (TB) is emerging as a top global health priority, after years of being overshadowed by attention toward adult TB interventions [1, 2]. Compared to adult TB, TB in children under age 15 presents symptoms that are difficult to detect [3, 4]. Confirming diagnoses bacteriologically is a challenge, as children are often unable to expectorate sputum, and typically present as sputum smear-negative due to low bacillary loads [3, 5]. Diagnostic tools such as GeneXpert MTB/RIF (Cepheid, USA) assay and chest X-rays have been found to aid in diagnosis [3, 6], but in resource-poor health facilities with limited access to diagnostic tools, diagnosis becomes an even greater challenge [7]. In the absence of diagnostic tools and the ability to collect sputum, a successful childhood TB diagnosis requires the knowledge and discretion of the clinician to diagnose based on signs, symptoms, and contact history, assessed during clinical examination [8].

There is a distinct gap in the literature on how childhood TB is diagnosed in resource-poor settings, and the quality of these diagnoses. A 2015 *Lancet Respiratory Medicine* news article describes a pilot project to train health workers in Uganda, a high-burden TB country, on how to conduct symptom-based childhood TB diagnoses, using whatever equipment is available [9]. The article notes that barriers include insufficient health worker knowledge, and lack of diagnostic equipment [9]. Similarly, a 2015 qualitative study conducted in Peru noted that barriers to diagnosing childhood TB included “limited access to diagnostic tests,” and “inadequately trained health center staff” [10].

Cambodia, a low-income country, is one of the 22 countries with the highest TB burdens in the world, with a TB incidence rate of 390 per 100,000 population [11]. In 2013, the National Tuberculosis Programme (NTP) implemented routine services for childhood TB in 13 out of the 24 provinces in the country [12]. Diagnosing childhood TB remains a persistent challenge, however. While childhood TB is estimated to make up 10–20% of total TB cases in high-burden settings [1], this proportion ranges from 1.3 to 39.4% throughout Cambodia’s 24 provinces, suggesting potential under- and over-diagnosis of childhood TB, subnationally [12]. Notably, the proportion of case notifications classified as extrapulmonary (EPTB) out of total case notifications in children is 87% [12], greatly exceeding the expected global range of 20–30% [1]. These statistics reveal the need to examine how childhood TB diagnoses are being made, particularly under the constraints of limited resources and diagnostic tools.

The aim of the present study is to quantitatively assess the quality of childhood TB diagnoses in high-burden districts in Cambodia, by determining availability and usage of diagnostic tools, and clinician knowledge and practice. Recommendations are provided with the goal of improving effectiveness and safety in the diagnosis of childhood TB. Thus far, no study has quantitatively assessed the quality of childhood TB diagnoses in Cambodia. In the face of limited data, results from the present study could shed light on potential solutions to improve the quality of childhood TB diagnoses both in Cambodia, and other resource-poor, high-burden nations.

## Methods

Cambodia’s provinces are broken up into Operational Districts (ODs) that cover an average of 180,000 people, providing health care and TB services through referral hospitals and health centers [13]. ODs manage and report all quarterly TB case notifications to the NTP [14]. Referral hospitals are the primary location where children receive TB diagnoses; as such, they were chosen as health facilities of interest for the study.

### Study design and population

Between August and September of 2015, a cross-sectional study was conducted at referral hospitals and villages in the top 24 high-burden ODs. High-burden ODs were defined as ODs with a population above 100,000, and 25% or higher childhood TB as a proportion of all TB cases. A team of trained interviewers conducted interviews with clinicians from each referral hospital to determine clinician knowledge of childhood TB, availability and usage of diagnostic tools, and perceptions on capacity building. Medical and laboratory records of the utilization of diagnostic tools at health facilities were either not systematically recorded or unavailable; therefore, to cross-check clinician data, another team of trained interviewers traveled to nearby village homes to interview parents/guardians (referred to as “parents”) of children who had been recently diagnosed with TB and started treatment within the past three months.

The selection of high-burden ODs for the study took into account the percentage of childhood TB out of all TB cases for each OD, as well as OD population size, to ensure that a wide population was covered: ODs were sorted by population size in 2014 and assigned a numerical population ranking (a ranking of 1 indicated the largest population size); ODs were then sorted by percentage of childhood TB out of all TB cases in 2014 and assigned a numerical childhood TB ranking (a ranking of 1 indicated the largest percentage of childhood TB). We then averaged the two numerical rankings for each OD, and sorted ODs by this Average Rank number. The top 24 Average Rank numbers were the ODs selected for the study.

**Fig. 1:**
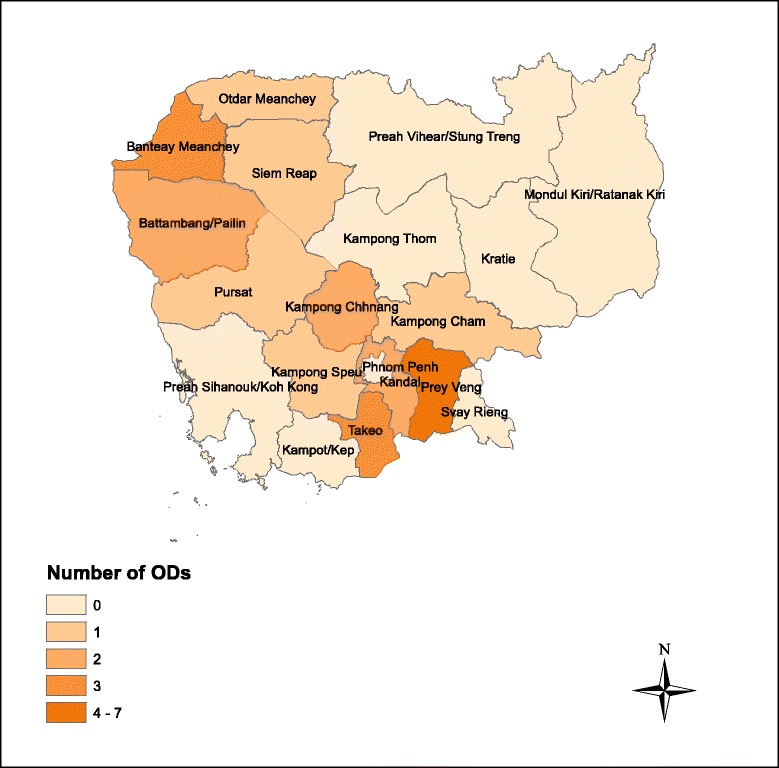
Geographical Distribution of Number of Operational Districts by Province/Region

Figure 1 displays the geographical distribution of the number of ODs selected by province/region across the country. These high-burden ODs tend to fall under regions with higher population density and higher sputum-positive TB case notifications [13]. As such, the NTP focuses attention and resources toward these areas to train clinicians on how to diagnose childhood TB. Cambodia’s capital, Phnom Penh, is one exception. Phnom Penh has five ODs with relatively low percentages of childhood TB. As a result, none of these ODs were selected. These low percentages, however, may not be an accurate reflection of the true burden of childhood TB in the city. National statistics do not take into account case notifications from private hospitals, of which there are several in Phnom Penh that provide free TB services.

A total of 40 clinicians and 104 parents were interviewed. Selection of clinician participants was defined as the two clinicians or practitioners (referred to in this study as “clinicians”) that regularly diagnosed and treated childhood TB at the referral hospital. There were typically no more than two clinicians who diagnosed and treated childhood TB at each referral hospital; sometimes there was only one. Given this, our sample size of 40 clinicians was deemed sufficiently representative of the 24 high-burden OD referral hospitals. Parent participants were selected systematically as the parents or guardians of the four most recently diagnosed children, according to the referral hospital’s TB records. Interviewers visited parent participants at their homes, which varied in distance from the referral hospital: some homes were five minutes from the hospital, while others were as far as an hour away, including travel by ferry to reach river islands. Taking into account difficulty of travel and resource constraints, the sample size of four parent participants per hospital was deemed feasible for the study. Interviewers were able to reach four to five parent participants per hospital. If time allowed, a fifth parent participant was interviewed.

Permission to consult TB records to select parent participants was granted by administration at the referral hospital, though administration was not involved in the selection process. Participation was voluntary, and personal identifying information was not collected. The study received ethical approval from the National Ethics Committee for Health Research (NECHR) (reference number: 331 NECHR, Ministry of Health, Cambodia).

### Questionnaires

Interview questionnaires were designed based off of preliminary 2014 qualitative data (unpublished observations conducted by the WHO Cambodia’s Stop TB team). Qualitative data consisted of transcripts from focus groups and semi-structured interviews with health workers from five ODs, about diagnosing childhood TB. Questionnaire design was further informed by a literature review of knowledge, attitude, and practice-based questionnaires for physicians. TB content is based off of the National Guidelines for Diagnosis and Treatment of TB in Children [12], Cambodia’s clinical diagnostic algorithm, and The Union and WHO’s collaborative online course on childhood TB for health workers [8].

Questionnaires were written in English, and translated to Khmer. To minimize bias, interviewers were trained not to prompt the interviewee with the answers, but to let the interviewee respond freely. Questionnaires were pre-tested in a sub-sample of clinicians and parents to determine clarity and appropriateness of questions, and modified as needed.

#### Clinician questionnaire

The Clinician Questionnaire contained 23 questions, divided into four sub-categories: Demographics, Clinician Knowledge, Availability of Diagnostic Equipment, and Provider Perceptions on Capacity Building. Demographics assessed the age, gender, current position, and educational background of clinicians. In Cambodia, medical doctors (who undergo six years of education and two years of residency [15]), and specialists (doctors with three to four years of additional training) are the only medical personnel who are able to diagnose and treat TB. Medical assistants (who undergo a 12-month internship and training) and nurses (who undergo a 12-month program [15]) are able to screen and refer presumptive TB patients to doctors and specialists for diagnostic work ups; however, due to a shortage of physicians in Cambodia [15], medical assistants and nurses may take on a larger role at referral hospitals. Therefore, it is important to determine the educational background of clinicians interviewed, along with their current position.

The Clinician Knowledge section assessed clinician knowledge of key signs and symptoms of pulmonary and extra pulmonary childhood TB, using the clinical diagnostic algorithm for TB in children (typically posted on the walls of health facilities) and the National Guidelines for Diagnosis and Treatment of TB in Children [16] as benchmarks for measurement. Clinician knowledge of seven main screening criteria for childhood TB (including history, signs, and symptoms) was assessed: enlarged lymph nodes, persistent cough, persistent wheezing, child has PTB smear positive contact, weight loss/failure to gain weight, fever, and drenching night sweats.

The Availability of Diagnostic Equipment section assessed what diagnostic equipment was available, functional, and used to diagnose childhood TB at the referral hospital. Cambodia’s clinical diagnostic algorithm for pulmonary TB in children calls for sputum smear microscopy, and/or chest X-ray and TST if smear results are negative, sputum is not available, or the child has no cough. Referral hospitals are expected to have equipment for bacteriological confirmation (e.g. sputum smear microscopy), chest X-ray, and TST; however, preliminary 2014 qualitative data suggested that X-ray machines were often unavailable, broken, or not used because the clinician was not comfortable interpreting radiographic film. Qualitative data also suggested that sputum smear microscopy was often not conducted for children because children required gastric aspiration to induce sputum, and the proper equipment for gastric aspiration was not available.

The Provider Perceptions on Capacity Building section asked clinicians their opinions and perceptions on what could be done to improve the quality of childhood TB diagnoses at their facility, including more or improved training or equipment.

#### Parent questionnaire

The Parent Questionnaire consisted of nine brief questions to determine age and gender of the child, symptoms for which they sought treatment, and how clinicians conducted the clinical exam, to assess clinician performance. Due to concerns about the willingness of parent participants to disclose personal information given the stigma of a TB diagnosis in the family, characteristics of parents were not included in the Parent Questionnaire. Parent Questionnaires were designed to be as simple, brief, and minimally invasive to parents as possible, with cultural sensitivity as a top priority.

### Statistical analysis

Data entry was performed on EpiData version 3.1. Analyses were conducted using SAS version 9.4. Descriptive statistics were calculated to provide cross-sectional data. Any questions that participants chose not to answer were coded as “missing,” and were not included in the analysis. The total number of responses (“n”) to each question is included along with all percentages in the results below.

## Results

### Demographics

The 24 high-burden ODs selected were distributed throughout 11 provinces, displayed in Fig. 1. 40 clinicians and 104 parents were interviewed. Of the clinicians interviewed, 39 were male, and 1 was female (Table 1). The median clinician age was 45 years old (IQR: 42.5–48). 62.5% of clinicians reported that their highest educational degree attained was “Doctor,” while 22.5% reported “Medical Assistant,” 10% reported “Secondary Nurse,” 2.5% reported “Primary Nurse,” and 2.5% reported “Specialist” (*n =* 40). Of the 104 parents interviewed, 57 had a male child recently diagnosed with TB, and 47 had a female child. The median age of the recently diagnosed child was 7 (IQR: 5–10) (Table 1).

**Table 1:** Demographics of Clinicians and Parents

Clinicians	Frequency (*n =* 40)	Percent
Gender		
Male	39	97.5
Female	1	2.5
Median age, in years (IQR)	45 (42.5–48)	
Current Position		
Doctor	24	60.0
Medical Assistant	8	20.0
Nurse	4	10.0
Other	4	10.0
Highest Educational Degree Attained		
Primary Nurse	1	2.5
Secondary Nurse	4	10.0
Medical Assistant	9	22.5
Doctor	25	62.5
Specialist	1	2.5
Number of Years Diagnosing Childhood TB		
Less than 1 year	6	15.0
1–3 years	14	35.0
3–5 years	4	10.0
More than 5 years	16	40.0
Parents	Frequency (*n =* 104)	Percent
Gender of Child		
Male	57	54.8
Female	47	45.2
Median age of child, in years (IQR)	7 (5–10)	

### Clinician knowledge

In response to the question, “Please name the screening criteria for childhood TB (including history, signs, and symptoms),” 100% of clinicians correctly named “Enlarged lymph nodes,” 85% of clinicians correctly named “Persistent cough,” 97.5% of clinicians correctly named “weight loss/failure to gain weight,” 92.5% of clinicians correctly named “Fever,” 70% correctly named “Child has PTB smear positive contact,” and 50% correctly named “Drenching night sweats” (*n =* 40) (Table 2). In total, 95% of clinicians correctly answered 4 out of 7 main TB screening criteria, and 72.5% correctly answered 5 out of 7 (*n =* 40).

**Table 2:** Clinician Responses to the Question: “Please name the screening criteria for childhood TB”^a^

Criteria Named by Clinician^b^	Frequency (*n =* 40)	Percent
Enlarged lymph nodes	40	100.0
Persistent cough	34	85.0
Persistent wheezing	5	12.5
Child has PTB smear positive contact	28	70.0
Weight loss/failure to gain weight	39	97.5
Fever	37	92.5
Drenching night sweats	20	50.0

^a^Includes history, signs, and symptoms

^b^All criteria listed were deemed acceptable answers.

In follow-up questions, 86.8% of clinicians (*n =* 38) correctly answered that the duration of a persistent cough for TB should be for “2 weeks or more” (Table 3). 82.5% of clinicians (*n =* 40) correctly answered that lymph nodes should be enlarged “2 cm or more” to be characteristic of TB, and 85% correctly answered at least three characteristics of enlarged lymph nodes that imply TB (*n =* 40). 80.7% of clinicians (*n =* 31) said that they would treat the patient with antibiotics first if they were unsure whether the patient had TB.

**Table 3:** Clinician Responses to Follow-Up Knowledge Questions on Childhood TB Symptoms

Questions	Frequency	Percent
How long should persistent cough last?	*n =* 38	--
Correctly answered “2 weeks or more”	33	86.8
Answered incorrectly	5	13.2
How enlarged should the lymph node be?	*n =* 40	--
Correctly answered “2 cm or more”	33	82.5
Answered incorrectly	7	17.5
What characteristics of lymph nodes imply TB?	*n =* 40	--
Correctly named at least 3 characteristics	34	85
Unable to name 3 characteristics	6	15
If unsure of diagnosis, would you put patient on antibiotics first or start TB treatment?	*n =* 31	--
Antibiotics	25	80.7
TB treatment	2	6.5
Other	4	12.9

### Availability of diagnostic equipment

Clinicians were asked what equipment was available, functional, and used to diagnose (or assist in the diagnosis of) childhood TB at their health facility out of a list of diagnostic equipment. In response, 87.5% of clinicians reported “Stethoscope,” 70% reported “Thermometer,” 77.5% reported “Chest X-ray,” 65% reported “Sputum smear microscopy,” 7.5% reported “TB culture,” 70% reported “TST,” 27.5% reported “Xpert,” 5% reported “Equipment to perform gastric aspiration,” 7.5% reported “Equipment to perform sputum induction,” 10% reported “Equipment to perform lymph node aspiration biopsy,” and 7.5% listed “Other – Echography” (*n =* 40) (Table 4).

**Table 4:** Availability of Diagnostic Equipment in Health Facilities, as Reported by Clinicians

Available Diagnostic Equipment	Frequency (*n =* 40)	Percent
Stethoscope	35	87.5
Thermometer	28	70.0
Chest X-ray	31	77.5
Sputum smear microscopy	26	65.0
TB culture	3	7.5
TST	28	70.0
Xpert	11	27.5
Equipment to perform gastric aspiration	2	5.0
Equipment to perform sputum induction	3	7.5
Equipment to perform lymph node aspiration biopsy	4	10.0
Other - Echography	3	7.5

### Provider perceptions on capacity building

Clinicians were asked what the National TB Programme could do to help improve diagnosing childhood TB at their health facility: 92.5% of clinicians stated “more training,” and 85% stated “more or improved equipment” (*n =* 40). Those who said “more or improved equipment” were asked which equipment: 64.7% requested chest X-rays, 52.9% requested TST, 44.1% requested Xpert, and 44.1% requested equipment to perform gastric aspiration (*n =* 34). Those who said “more training” were asked what type of training would be useful. 86.5% requested training on how to diagnose childhood TB, and 86.5% requested training how to use diagnostic tools (*n =* 37). Those who requested training on how to use diagnostic tools were asked which diagnostic tools: 90.6% answered chest X-ray, and 62.5% answered TST (*n =* 32).

## Parent report of child’s signs and symptoms

Parents were asked what symptoms their child had prior to the TB diagnosis, for which they sought treatment. In response, 89.4% of parents stated that their child had an “enlarged lymph node,” and 27.9% said that their child had a “persistent cough” (*n =* 104). Of those who said that their child had an “enlarged lymph node,” 60.2% named characteristics of the enlarged lymph node that did not meet criteria suggestive of TB (lymph nodes that were painful, less than 2 cm, and/or had persisted for less than 2 weeks prior to TB diagnosis) (*n =* 93). Of those that described lymph nodes that were not suggestive of TB, 35.9% said that the doctor did not first put the child on another drug to see if that would work, before starting TB treatment (*n =* 53).

### Parent report of clinical assessment

Parents were asked what examinations and tests the doctor did during the clinical assessment of the child: 56.7% of parents reported “TST,” 44.2% reported “Lymph node palpation,” 34.6% reported “Chest X-ray,” 17.3% reported “Checked the child’s temperature,” and 11.5% reported “Listened to the child’s chest with a stethoscope” (*n =* 104) (Table 5). None of the parents reported that the clinician conducted sputum smear microscopy, TB culture, Xpert, gastric aspiration, sputum induction, or lymph node aspiration biopsy. Further analyzing this data, 89.4% of parents stated 2 or fewer examinations/tests that the clinician conducted (*n =* 104). The mean number of examinations conducted was 1.64 (median = 2, std dev = 0.99). 75% of parent responses consisted of “TST” and/or “palpation,” and only one other examination or test (*n =* 104).

**Table 5:** Examinations and Tests Conducted by Clinician During Clinical Assessment, as Reported by Parents

Examinations Conducted by Clinician	Frequency (*n =* 104)	Percent
Listened with stethoscope	12	11.5
Checked temperature	18	17.3
Chest X-ray	36	34.6
Sputum smear microscopy	0	0
TB culture	0	0
Xpert	0	0
Gastric aspiration	0	0
Sputum induction	0	0
Lymph node aspiration biopsy	0	0
TST	59	56.7
Palpation	46	44.2

## Discussion

### Demographics

The majority of clinicians interviewed were male, reflecting a male-dominated health workforce that is prevalent throughout the health system in Cambodia. In 2011, only 16% of doctors, 8% of specialists, 35% of primary nurses and 31% of secondary nurses were female [17]. The majority of clinicians interviewed stated that the highest educational degree they attained was “Doctor;” however, a total of 35% of clinicians had achieved an educational degree no higher than that of a medical assistant or nurse. Doctors and specialists are the only medical personnel qualified to diagnose and treat TB, yet there is a dire shortage of physicians in Cambodia. In 2015, there were only 1.7 physicians per 100,000 population in the country and 7.9 “nursing and midwifery personnel,” respectively [18]. The presence of under-qualified medical personnel regularly diagnosing and treating childhood TB in high-burden ODs suggests that some referral hospitals do not have a physician available to diagnose and treat childhood TB. This raises concerns about the capacity of these referral hospitals to provide quality care. The World Health Organization’s 2015 “Health System Review” of Cambodia notes that a shortage of qualified physicians is one of the major challenges for the health system [15]. Hospitals rely heavily on nurses to provide care, particularly in rural districts, but the quality of training and education nurses and other health workers receive has been called into question [15].

### Limited diagnostic technology

Availability of proper diagnostic tools across health facilities was low. None of the equipment listed was available at 100% of facilities, including basic equipment such as stethoscopes and thermometers. Equipment available at more than half of health facilities were: chest X-ray, stethoscope, thermometer, sputum smear microscopy, and TST. This leaves clinicians with few options for confirming childhood TB diagnoses through a reliable diagnostic tool. Chest X-rays can assist in diagnosing pulmonary TB (PTB) only if the clinician knows how to read and interpret radiographic film (and parent data suggests that X-rays are rarely used, which will be discussed further). Sputum smear microscopy is difficult to perform for children, as children often cannot produce sputum (therefore equipment for gastric aspiration or sputum induction needs to be available), and children typically present as sputum smear negative [3, 5]. TST determines when someone has been exposed to the infection, but it cannot discern whether someone has active TB. Thus, it is considered to be an adjunctive diagnostic tool, as specificity of a positive TST is suboptimal [19].

### Chest X-Ray usage

Although 77.5% of clinicians stated that they have a chest X-ray at their health facility, only 34.6% of parents reported that the clinician conducted a chest X-ray. There are a number of potential reasons for this gap between X-ray availability and usage: clinicians may know how to conduct X-rays and interpret film but choose not to (perhaps due to cost, or perceptions of inconvenience); clinicians may not know how to conduct X-rays or interpret film; or X-ray machines that are available may not work properly. A high percentage of clinicians requested more training on how to use X-rays and interpret radiographic film, (this included reading and interpreting radiographic film), as well as “more or improved” chest X-rays.

### Clinician knowledge and performance

Clinicians were able to name the majority of main screening criteria for childhood TB (although notably, 15% of clinicians missed “persistent cough,” one of the top symptoms of PTB), as well as a number of key symptom characteristics. However, results from the parent data suggest that in practice, clinicians may not be applying their knowledge to a clinical exam. Although 92.5% of clinicians named “fever” as a screening criterion, only 17.3% of parents reported that the clinician checked the child’s temperature during the exam. The majority of clinicians could cite that lymph nodes should be enlarged 2 cm or more, and could name at least three characteristics of enlarged lymph nodes that imply TB; however, 60.2% of parents whose child had an enlarged lymph node described lymph node characteristics that were not suggestive of TB.

Few clinicians were reported to have listened to the child’s chest with a stethoscope during the clinical assessment. The average number of examinations conducted during the clinical assessment was less than 2, which would be inadequate under any clinical setting. The ability to apply knowledge to clinical practice is imperative for an accurate child TB diagnosis.

### Clinician knowledge of EPTB

The most common manifestation of EPTB is enlarged lymph nodes, or lymphadenitis [20]. Globally, EPTB makes up 20-30% of all childhood TB cases [1]. Given that lymphadenitis is a symptom of many ailments common in children, if a child presents with swollen lymph nodes it is important not to assume that it is EPTB, and conduct a full and careful clinical assessment [20]. Ideally, a lymph node aspiration biopsy would be performed [20]; however, availability of the necessary equipment was quite low.

Palpation was frequently one of the few examinations or tests clinicians conducted before diagnosing the child with TB. 89.4% of parents described “enlarged lymph node(s)” as a symptom that their child had, for which they sought treatment. The majority of parents whose child had “enlarged lymph node(s)” described lymph node characteristics that did not imply TB, but the child was still diagnosed with TB and put on treatment, usually without a trial of any other drug beforehand. This data raises concern about suboptimal clinical assessment, and rapid diagnoses based on “enlarged lymph node(s)” that may not have characteristics that imply TB. The danger of diagnosing TB without diagnostic confirmation and certainty is that misdiagnosed children will undergo an unnecessary, arduous 6-month TB treatment regimen, with many side effects [21]. Further research should be conducted to determine whether inaccurate EPTB diagnoses could partially explain the unusually high proportion of EPTB out of all TB cases in children.

### Recommendations

Clinician training for diagnosing childhood TB should be revamped to emphasize how to conduct a clinical exam, assess symptoms, and diagnose without the availability of advanced diagnostic tools (The Union and WHO’s Childhood TB Learning Portal has many applicable examples [8]). Clinician training should also emphasize the practical application of knowledge as opposed to recall and memorization of facts. One way to do this would be through the standardized patient approach [22–24], and simulating a clinical exam during routine on-site supervision. Clinician training should additionally emphasize that “enlarged lymph node” is a common symptom of many ailments – if the child with an enlarged lymph node does not have any other suggestive indications of EPTB, a 10-day (minimum) trial of antibiotics (excluding fluoroquinolones, due to their anti-TB properties [25, 26]) should be given with a follow-up.

Resources for diagnostic tools should prioritize well functioning chest X-ray machines, and training for how to use the machines and interpret radiographic film. Equipment for bacteriological confirmation and gastric aspirates should be available at all health facilities, with training for how to use the equipment. For facilities that do not have access to chest X-rays or equipment for bacteriological confirmation, transport should be available to facilities that do. Basic equipment such as stethoscopes and thermometers should be available at 100% of facilities.

### Limitations

One limitation of the study is that parent interviews are an imperfect proxy for how clinicians conduct exams, and may present biases: Parents may not have remembered everything that the clinician did during the clinical assessment, or every symptom their child had, and responses may differ depending on the personal characteristics of the parent or the clinician that they saw. In an effort to minimize recall bias, only parents of children who were diagnosed within the past three months were interviewed. Additionally, the small sample size of clinicians, though representative of referral hospitals in the 24 high-burden ODs, is not representative of referral hospitals in low-burden ODs. Further research should be conducted to determine capacity of clinicians and health facilities in low-burden ODs, where attention and resources toward addressing childhood TB are even lower.

## Conclusion

Diagnosing childhood TB is difficult, but especially so in resource-poor nations such as Cambodia. The present study provides insight into the capacity of Cambodian hospitals to diagnose childhood TB in high-burden districts, and the challenges of diagnosing childhood TB with constrained resources. Limited availability of diagnostic tools and suboptimal clinician performance highlights where resources need to be allocated to improve the quality of diagnoses. It also serves as an indicator for the quality of TB services at referral hospitals for both childhood and adult TB. Further research needs to be done in low-burden ODs to determine the capacity of clinicians and health facilities for diagnosing childhood TB, where cases are likely being missed. Low-burden ODs, particularly those in rural settings, may face even greater challenges for diagnosing childhood TB, including fewer resources. Improving health facilities’ data collection and records would facilitate future empirical research, providing researchers with a clearer understanding of the quality of both childhood and adult TB diagnoses.

Few studies have explored childhood TB diagnosis in resource-poor settings. By illuminating the current state of childhood TB diagnosis in high-burden districts in Cambodia, the present study provides some of the first evidence of the capacity of resource-poor health facilities to diagnose TB in children. Results and recommendations from this study may be useful for other National TB Programs and Ministries of Health trying to determine where and how to allocate resources to improve the quality of childhood TB diagnoses. Highlighting some of the challenges faced in Cambodia places a much needed spotlight on childhood TB, and may provide a framework for other settings to take a closer look at the current state of childhood TB diagnostic practices in their healthcare system. Results from this study can inform evidence-based policy both in Cambodia and other resource-poor nations, and serve as a building block for future research and assessment.

## Abbreviations

EPTB: Extrapulmonary tuberculosis
NECHR: National Ethics Committee for Health Research
NTP: National Tuberculosis Programme
OD: Operational District
PTB: Pulmonary tuberculosis
TB: Tuberculosis
WHO: World Health Organization
